# The root-knot nematode *Meloidogyne incognita* produces a functional mimic of the Arabidopsis INFLORESCENCE DEFICIENT IN ABSCISSION signaling peptide

**DOI:** 10.1093/jxb/ery135

**Published:** 2018-04-10

**Authors:** Joonyup Kim, Ronghui Yang, Caren Chang, Younghoon Park, Mark L Tucker

**Affiliations:** 1 Soybean Genomics and Improvement Laboratory, Agricultural Research Service, USDA, Beltsville, MD, USA; 2 Department of Cell Biology and Molecular Genetics, Bioscience Research Bldg, University of Maryland, MD, USA; 3 Life and Industry Convergence Research Institute, Department of Horticulture Bioscience, Pusan National University, Miryang, Republic of Korea

**Keywords:** Abscission, Arabidopsis, IDA, *Meloidogyne incognita*, MiIDL1, peptide signal, RNAi, root-knot nematode

## Abstract

INFLORESCENCE DEFICIENT IN ABSCISSION (IDA) is a signaling peptide that regulates cell separation in Arabidopsis including floral organ abscission and lateral root emergence. IDA is highly conserved in dicotyledonous flowering plant genomes. *IDA-like* sequences were also found in the genomic sequences of root-knot nematodes, *Meloidogyne* spp., which are globally deleterious pathogens of agriculturally important plants, but the role of these genes is unknown. Exogenous treatment of the Arabidopsis *ida* mutant with synthetic peptide identical to the *M. incognita* IDA-like 1 (MiIDL1) protein sequence minus its N-terminal signal peptide recovered both the abscission and root architecture defects. Constitutive expression of the full-length *MiIDL1* open reading frame in the *ida* mutant substantially recovered the delayed floral organ abscission phenotype whereas transformants expressing a construct missing the MiIDL1 signal peptide retained the delayed abscission phenotype. Importantly, wild-type Arabidopsis plants harboring an *MiIDL1*-RNAi construct and infected with nematodes had approximately 40% fewer galls per root than control plants. Thus, the *MiIDL1* gene produces a functional IDA mimic that appears to play a role in successful gall development on Arabidopsis roots.

## Teaser

A root-knot nematode gene (*MiIDL1*) produces a peptide that mimics the Arabidopsis IDA signaling peptide that regulates cell separation events including abscission and lateral root emergence.

## Introduction

Abscission is the process by which organs (leaves, flowers, fruit, petals, stems, etc.) are shed from the parent plant (Roberts *et al.*, 2002; Estornell *et al.*, 2013). An innate part of abscission is cell separation, which is essential for additional developmental processes including organogenesis, emergence of lateral roots, pollen tube elongation through the pistil, seed germination, and pod and anther dehiscence (Roberts *et al.*, 2002). A part of all cell separation events is the decomposition of the middle lamella that glues cells together and, very often, a loosening or restructuring of the primary cell wall (Roberts *et al.*, 2002).

In 2003, Butenko *et al.* (2003) described an *Arabidosis thaliana* mutant *inflorescence deficient in abscission* (*ida*) that does not abscise (drop) its floral organs. The *IDA* gene encodes a small protein of 77 amino acids that includes an N-terminal signal peptide for secretion into the apoplast. IDA is a member of a small gene family in Arabidopsis that encodes proteins with a highly conserved proline-rich, C-terminal sequence of 12 amino acids. Since its nascent discovery, an understanding of the IDA signaling pathway and mode of action have progressed considerably (Cho *et al.*, 2008; Stenvik *et al.*, 2008*b*; Butenko and Simon, 2015; Vie *et al.*, 2015; Meng *et al.*, 2016; Santiago *et al.*, 2016). The IDA protein is secreted into the apoplast where it is cleaved into a smaller peptide (Stenvik *et al.*, 2008*b*; Butenko and Simon, 2015; Schardon *et al.*, 2016). It was demonstrated that the previously identified, conserved 12 amino acid C-terminal sequence of IDA was sufficient to rescue the delayed abscission phenotype of *ida*, but a slightly longer 14 amino acid fragment of the C-terminus induced a higher percentage of abscission at a lower concentration (Schardon *et al.*, 2016). The resultant mature IDA peptide binds to two redundant leucine-rich receptor-like kinases HAESA (HAE) and HAESA-like 2 (HSL2) localized in the plasma membrane (Cho *et al.*, 2008; Stenvik *et al.*, 2008*a*; Santiago *et al.*, 2016). Recent studies demonstrate that another class of receptor-like kinases, SOMATIC EMBRYOGENESIS RECEPTOR KINASE (SERK), facilitates binding of IDA to HAE and HSL2 (Meng *et al.*, 2016; Santiago *et al.*, 2016), which then relay the binding signal through a MAP kinase phosphorylation signaling pathway that ultimately activates a set of KNOX transcription factors that regulate a change in gene expression. It was proposed that the role of IDA in abscission is to regulate gene expression for cell wall modifying proteins, e.g. polygalacturonases (PGs), expansins (EXPs), cellulases, xyloglucan endotransglucosylase/hydrolase (XTH) and others (Butenko *et al.*, 2003; Shi *et al.*, 2011).

Because of the proposed role for IDA in regulating genes for cell wall modification, it was suggested that IDA might regulate other cell separation events in Arabidopsis (Butenko *et al.*, 2003). Subsequently, IDA signaling was demonstrated to be involved in the emergence of lateral roots through the root cortex (Kumpf *et al.*, 2013). In lateral root emergence, the HAE and HSL2 receptors each appear to play a role at a different stage of root emergence. Comparison of gene expression patterns for cell wall modifying proteins including PG LATERAL ROOT (PGLR), PG ABSCISSION ZONE ARABIDOPSIS THALIANA (PGAZAT/ADPG2), EXPANSIN 17 (EXP17) and XYLOGLUCAN TRANSFERASE 6 (XTR6/XTH23) in the wild-type, *ida* and the *hae*/*hsl2* double mutant indicated that expression of these genes during lateral root emergence increased in an IDA signaling-dependent manner (Kumpf *et al.*, 2013).

More recently, it was reported that *IDA-like* genes, *AtIDL6* and *AtIDL7*, play a role in moderating stress and defense responses in Arabidopsis (Vie et al., 2015, 2017; Wang *et al.*, 2017). It was demonstrated that *AtIDL6* and *AtIDL7* were strongly up-regulated by treatment with the bacterial flagellin elicitor, flg22, and infection with *Pseudomonas syringae* (Vie *et al.*, 2015, 2017; Wang *et al.*, 2017). An elevated expression of *IDL6* during infection by *P. syringae* increased susceptibility to the pathogen (Wang *et al.*, 2017). It was proposed that *IDL6* increased the expression of poylgalacturonases that enhanced infection by *P. syringae*. Moreover, the authors noted an *IDL6*-dependent suppression of the *PATHOGENESIS-RELATED PROTEIN-1* gene, *PR1*. Also, of interest was that the IDL6 response was at least partially dependent upon the presence of the *HAE* and *HSL2* receptors. It was subsequently demonstrated that both *IDL6* and *IDL7* suppressed several stress- and defense-associated genes, and that *IDL7* and, maybe *IDL6*, reduced stress-induced reactive oxygen species (ROS) signaling (Vie *et al.*, 2017).

Genes encoding IDA-like (IDL) proteins are conserved in land plants (Tucker and Yang, 2012; Estornell *et al.*, 2015; Stø *et al.*, 2015; Vie *et al.*, 2015). *IDA-like* genes have been found in dicots, monocots, and some gymnosperms (Stø *et al.*, 2015; Vie *et al.*, 2015). Of interest is that the conserved C-terminal domain of IDA-like proteins shares sequence similarity with the conserved C-terminus of CLAVATA3 (CLV3) (Stenvik *et al.*, 2008*b*; Butenko and Simon, 2015) and PAMP-INDUCED PEPTIDES (PIP) (Vie *et al.*, 2015). CLV3 is a small, secreted peptide that is transcribed and translated in the central zone of the shoot apical meristem where it is secreted and then processed in the apoplast (Barton, 2010). CLV3 binds to leucine-rich repeat receptor-like kinase proteins (LRR-RLK) CLAVATA1 and CLAVATA2 in the plasma membranes of cells immediately below the undifferentiated stem cells where CLV3 is synthesized (Barton, 2010). The role of CLV3 secretion and feedback regulation from affected cells is to limit cell divisions in the shoot apical meristem (Barton, 2010). It has been proposed that IDA may have evolved from the more primal CLV3 protein to fulfill a different role in regulating plant development (Butenko and Simon, 2015). Thus, although IDA and CLV3 (CLAVATA3/ESR-related or CLE) proteins share sequence similarity (Butenko and Simon, 2015), IDA and CLE bind to different LRR receptor-like proteins and are functionally distinct (Guo *et al.*, 2010; Hou *et al.*, 2014; Butenko and Simon, 2015; Santiago *et al.*, 2016). *PIP1* and *PIP2*, which are the progenitors for a larger gene family, were first identified as being up-regulated by the pathogen-associated molecular pattern (PAMP) bacterial flagellin (flg22) (Hou *et al.*, 2014). Similar to CLV3, PIP1 and PIP2 are secreted into the apoplast where they are cleaved into smaller peptides that bind to the RECEPTOR-LIKE KINASE 7 (RLK7), which activates a plant immune response (Hou *et al.*, 2014).

Root-knot and cyst nematodes are pandemic pathogens that parasitize the roots of many agriculturally important plant species (Williamson and Hussey, 1996; Jung and Wyss, 1999; Hewezi and Baum, 2013; Mitchum *et al.*, 2013; Goverse and Smant, 2014; Hewezi, 2015). Cyst nematodes (*Heterodera* spp. and *Globodera* spp.) migrate through the root and attach to a pericycle or procambial cell in the vascular bundle to establish a feeding structure (syncytium) by secreting proteins (effectors) that alter root cell development and inhibit host defense responses (Hewezi and Baum, 2013; Goverse and Smant, 2014; Hewezi, 2015). The syncytium for cyst nematodes is formed by degrading the cell walls and membranes between existing root cells to form one large multinucleated cell that can eventually include as many as 200 host cells (Jung and Wyss, 1999). Root-knot nematodes (*Meloidogyne* spp.), on the other hand, migrate through the root tip to a parenchymatous cell in the still developing vascular bundle where they then become sessile and secrete effectors to establish their feeding structure (Hewezi and Baum, 2013; Mitchum *et al.*, 2013; Goverse and Smant, 2014). The root-knot nematode forms its feeding structure by inducing cell division without cytokinesis to produce several large multinucleated giant cells (Jung and Wyss, 1999). The giant cells are surrounded by more dividing cells that form a protective gall around the nematode and giant cells (Jung and Wyss, 1999).

Numerous effector proteins have been identified in both cyst and root-knot nematodes. Of these effectors, it has been shown that a few have similarity to signaling peptides found in the plant (Gheysen and Mitchum, 2011; Mitchum *et al.*, 2013; Goverse and Smant, 2014). For example, a CLV3-like (CLE) protein was one of the earliest effectors identified in the secretory esophageal glands of cyst nematodes (*Heterodera* spp.; Olsen and Skriver, 2003). Although it has been clearly demonstrated that the nematode CLE proteins play a role in establishing the syncytium (Hewezi, 2015) and that the nematode CLE peptide signals through a receptor complex that includes CLV1, CLV2 and RPK2 (Replogle *et al.*, 2011; Replogle *et al.*, 2013), the actual mechanism of action of the nematode CLE peptides that is needed for formation of the nematode feeding site is still being worked out (Guo *et al.*, 2015, 2017). Interestingly, the CLE motif is also found in root-knot nematodes; however, it exists as multiple tandem repeats of CLE-like motifs within a single gene (Rutter *et al.*, 2014), which was named *MAP* (*Meloidogyne Avirulence Protein*). The MAP protein is secreted from the nematode (Semblat *et al.*, 2001; Vieira *et al.*, 2011), but it is unknown if the larger MAP protein is processed into functional CLE peptides (Rutter *et al.*, 2014); nevertheless, similar to the root-knot nematode, the potato cyst nematode (*Globodera rostochiensis*) also possesses *CLE-like* genes with multiple CLE motifs within the same gene and these gene products are correctly processed into functional CLE-like peptides that rescued the Arabidopsis *CLV3* mutant, *clv3-2* (Guo *et al.*, 2011).

The proposed role for IDA in regulating gene expression for cell separation led us to hypothesize that IDA or an IDA-like peptide native to the plant (host) or nematode might play a role in dissolution and/or modification of the cell walls during formation of the syncytia or giant cells of cyst or root-knot nematodes, respectively. Quantitative PCR (qPCR) of RNA from soybean cyst nematode (SCN, *Heterodera glycines*)-infected soybean roots and southern root-knot nematode (*Meloidogyne incognita*)-infected tomato roots did not indicate a significant change in a plant *IDA-like* gene (Tucker and Yang, 2012, 2013) that was associated with the infection (unpublished results). Moreover, no *IDA-like* genes were found in the sequence data for cyst nematodes (*Heterodera* spp.); however, we did discover *IDA-like* genes in root-knot nematodes (*Meloidogyne* spp.; Tucker and Yang, 2013). *M. incognita* has two *IDA-like* genes, *MiIDL1* and *MiIDL2*, and the open reading frames (ORFs) of both genes contain a sequence for an N-terminal secretion peptide and are expressed early in the infection and development of galls on tomato roots (Tucker and Yang, 2013). Although the C-terminus of the *MiIDL1* and *MiIDL2* translational products are similar to that of *IDA* and *IDA-like* genes found in plants, we had no proof that the nematode *IDA-like* genes produce a functional IDA peptide. Herein, we demonstrate that the *MiIDL1* gene product can complement the *ida* mutant in Arabidopsis and thereby is capable of functioning as an IDA signal, which can bind to the receptor-like kinases such as HAE and HSL2 in Arabidopsis. In addition, we used an RNA interference (RNAi) approach to suppress *MiIDL1* expression in the nematode (Dutta *et al.*, 2014) and demonstrated that expression of the *MiIDL1*-RNAi construct in Arabidopsis roots significantly correlated with a reduction in the number of nematode infections (galls) that formed on the roots.

## Materials and methods

### Plant materials and growth conditions

Arabidopsis (*Arabidopsis thaliana*) seeds of Columbia (Col-0), C24, *ida*-*2* (Col-0 background), *ida-1* (C24 background) and the *hae-3*/*hsl2-3* (Col-0 background) double mutant were surface sterilized by treatment with 20% (v/v) bleach for 2 min followed by 70% ethanol for 5 min and then rinsed several times with sterile distilled water. After stratification at 4 °C for 2–4 d, sterilized seeds were geminated on agar plates containing 0.8% (w/v) agar, half-strength Murashige and Skoog basal salt mixture (Murashige and Skoog, 1962), pH 5.7. Plants used for peptide assays were grown on agar plates with or without synthetic peptides as described. For other experiments, after seed germination on agar plates, plants were transferred to Promix BX potting soil (Griffin Greenhouse Supplies, Richmond, VA, USA). Both plants on agar and plants in soil were grown in a growth chamber set to 23 °C, 16 h light (90–150 μE m^−2^ s^−1^), 8 h dark and 50–60% humidity.

### Retrieval of IDL and similar sequences and peptide alignment

The soybean (*Glycine max*) and tomato (*Solanum lycopersicum*) *IDA-like* sequences were those previously identified by a TBLASTN search of soybean and tomato genomic sequence data using the AtIDA (accession NP564941) ORF minus the predicted N-terminal signal peptide (Tucker and Yang, 2012). Additional genomes were later searched for IDA-like peptides using only the AtIDA EPIP domain (Stenvik *et al.*, 2008*b*). Other genes encoding similar C-terminal protein sequences, *CLV3*, *CLE-like*, *PIP*, *PIPL*, *MiMAP*, and *Mi16D10*, were selected from the literature and downloaded from the National Center for Biotechnology Information (NCBI). Alignments were performed using T-Coffee in the MacVector software (version 15.1). A dendrogram was created using UPMGA, best-tree with distances uncorrected. These settings were selected to align sequences based simply on identity and chemically similar amino acids and not a predicted evolutionary relationship. When a conserved C-terminal domain was not predicted in the literature, the sequence was first aligned with predicted domains and the most similar C-terminal sequence used in subsequent alignments. The accession numbers for the nucleotide and protein sequences are listed in Supplementary Table S1 at *JXB* online. Where an accession number was not available, the nucleotide and protein sequence is included in Supplementary Table S1.

### Peptide synthesis and mutant complementation assays

The peptides used in this study for complementation of the *ida* mutant were synthesized by Life Technologies (now Thermo Fisher Scientific, Waltham, MA, USA) at 90% purity (Table 1). Synthetic peptides were suspended in 1/10 volume of sterilized NaH_2_PO_4_ (0.125 M, pH 7.0) buffer and then transferred to 9/10 volume of ddH_2_O. For the floral organ abscission assay, Arabidopsis flowers were collected at anthesis from plants of wild-type (Col-0 and C24), *ida-1* (C24), *ida-2* (Col-0) and the *hae*/*hsl2* double mutant, *hae-3*/*hsl2-3* (Col-0) (abbreviated herein as *hh33*), and the pedicel of each flower was inserted into agar plates containing 10 μM of the corresponding peptides. To measure the percentage of abscission, individual flowers were touched with forceps and the numbers of flowers displaying petal separation were recorded after 48 h of treatment. Three to five biological replicates were performed for each peptide rescue assay and each assay included between 10 and 30 flowers. To assess the physiological response of Arabidopsis roots to the synthetic peptides, wild-type (Col-0) and *ida-2* seeds were grown for 48 h on agar plates containing 0, 0.1, 0.5, and 1.0 μM of the MiIDL1p peptide and the root development recorded.

**Table 1.** Sequence of synthetic peptides used in this study

Peptide name	Sequence
MiIDL1p	IKGVPPNSGPSRRGNKVPGPGR
AtIDAp	FGYLPKGVPIPPSAPSKRHN
GmIDA1ap	FNFLPKGVPIPPSGPSKRHN
AtCLV3p	LRTVPSGPDPLHHH
Mi16D10p	GKKPSGPNPGGNN

### Nematode culture and infection assay

Root-knot nematodes, *Meloidogyne incognita*, were maintained on the roots of pepper (*Capsicum annuum*) in a greenhouse at the Soybean Genomics and Improvement Laboratory, United States Department of Agriculture, Beltsville, MD, USA. *Meloidogyne incognita*-infected roots were harvested from pepper plants 3–4 months after inoculation. To isolate second-stage juveniles (J2), roots with galls were collected and shaken in 15% bleach for 5 min to release the eggs, and the eggs isolated by successively passing the liquid through stainless steel sieves with pore sizes of 850, 150, and 25 μm (no. 20, no. 150, and no. 500 sieves from Newark Wire Cloth, Clifton, NJ, USA; Meyer *et al.*, 2000). Eggs were collected from inside the no. 500 sieve and hatched in 100 ml of water with 50 μg ml^−1^ of ampicillin shaken at 28 °C. After 3–4 d hatching, J2 were isolated from the eggs using the no. 500 sieve. Arabidopsis transgenic seedlings were germinated on agar plates containing kanamycin 50 μg ml^−1^ for 10 d and transferred to 3.8 cm pots containing 90% sand 10% clay soil (Xue *et al.*, 2013). After 1 week of recovery in soil, approximately 1000 freshly hatched J2 were added to each pot. All J2-infected plants were fertilized with liquid Knops medium (Sijmons *et al.*, 1991) once a week for the first 2 weeks after transplanting. After 2, 3, 7, 14, 15, 35, 42, and 56 d post-inoculation (dpi), a sampling of infected roots of T3 transgenic plants was gently washed in tap water, blotted dry, and observed for a general assessment of infection. Based on our preliminary assessment of gall development and our ability to reproducibly count galls, a larger population of roots was harvested at 14, 35 and 42 dpi and the roots washed and stained using acid fuchsin as described by Bybd *et al.* (1983), which stains the nematodes red and enhances their detection. The number of stained galls per root was counted under a dissecting microscope.

### RNA isolation and qPCR

Total RNA was extracted from roots of root knot nematode-infected wild-type (Col-0) and transgenic plants using a TRIzol reagent (Invitrogen, Carlsbad, CA, USA). One microgram of total RNA was used for cDNA synthesis using Superscript-III™ Reverse Transcription System (Invitrogen), following the manufacturer’s instructions. The resultant cDNA was diluted 5-fold. Sequences of primers used for qPCR are listed in Supplementary Table S2. Gene-specific primers were used in a total volume of 15 μl consisting of 3 μl of diluted cDNA, 1.5 μl each of 1.0 μM gene-specific primers, 1.5 μl of ddH_2_O and 7.5 μl of Brilliant II SYBR Green QPCR Master Mix in an Mx3000P instrument (Stratagene, La Jolla, CA, USA). The qPCR results were normalized to either *MiEF1b* (Tucker and Yang, 2013) for the nematode genes or *AtACT2* (Cartieaux *et al.*, 2008) for the Arabidopsis genes and the transgenes β-glucuronidase (*GUS*), *MiIDL1*, or *NPTII*.

### Constructs and transgenic Arabidopsis that overexpressed MiIDL1 and MiIDL1-RNAi

For overexpression of MiIDL1 in plants, the ORFs with and without predicted signal peptide sequences of MiIDL1 (Tucker and Yang, 2013) were PCR amplified from genomic DNA of root-knot nematode by primers as described in Supplementary Table S2. Amplified PCR products were digested with *Xho*I and *Xba*I and then cloned into pHANNIBAL (Wesley *et al.*, 2001), which excised and replaced the *PYRUVATE ORTHOPHOSPHATE DIKINASE* (*PDK*) intron. The vector, pHANNIBAL, includes the CaMV 35S promoter for constitutive gene expression in plants. For root-knot nematode suppression assays, a 347 nt and 324 nt sense and antisense fragment of *E*. *Coli GUS* and root-knot nematode *MiIDL1*, respectively, were amplified using primers as described in Supplementary Table S2. The sense strand PCR product was introduced into the *Xho*I-*Eco*RI and antisense into *Xba*I–*Hin*dIII of pHANNIBAL on either side of the *PDK* intron, which generated the *GUS* and *MiIDL1*-RNAi (hairpin dsRNA) constructs. Both overexpression of *MiIDL1* and of *GUS* and *MiIDL1*-RNAi in the pHANNIBAL vector were digested with *Not*I, which included the 35S promoter and the *OCTOPINE SYNTHASE* (*OCS*) terminator, and cloned into pART27 (Wesley *et al.*, 2001). Plasmids pART27-*MiIDL1* (full length ORF, named MG) and pART27-*MiIDL1* (ORF minus signal peptide, named MS), pART27-*GUS*-RNAi (named DG), and pART27-*MiIDL1*-RNAi (named DM) were introduced into *Agrobacterium tumefaciens* GV3101 by electroporation, verified by PCR, and transformed into Arabidopsis *ida-2* (Col-0) and wild-type Col-0 plants by floral spray transformation (Chung *et al.*, 2000). Transgenic seeds were selected on half-strength Murashige and Skoog medium supplemented with kanamycin at 50 μg ml^−1^. Kanamycin-resistant plants were verified for expression of constructs and further used for the study as described.

## Results

### The nematode IDLs are more similar to the plant IDLs than to PIPs or CLEs

**Fig. 1.**
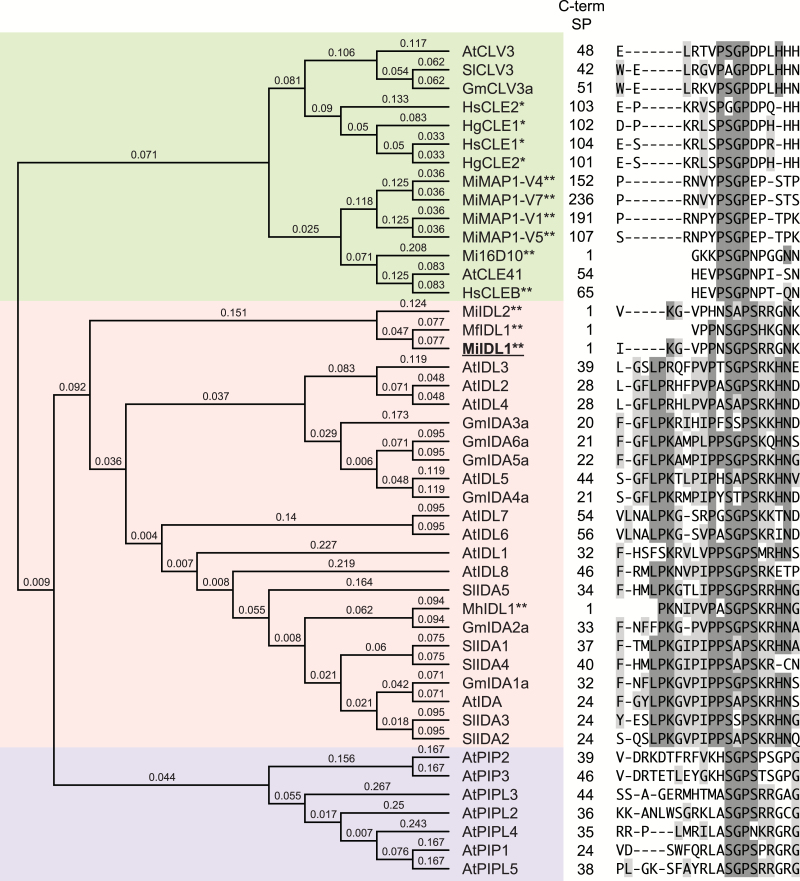
Dendrogram and alignment of conserved C-terminal amino acid sequences for a selection of representative plant and nematode genes with similarity to the AtIDA EPIP domain: Arabidopsis (*Arabidopsis thaliana*, At), soybean (*Glycine max*, Gm), tomato (*Solanum lycopersicum*, Sl), soybean cyst nematode (*Heterodera glycines*, Hg), sugar beet nematode (*H. schachtii*, Hs), southern root-knot nematode (*Meloidogyne incognita*, Mi), northern root-knot nematode (*M. hapla*, Mh) and peach root-knot nematode (*M. floridensis*). Sequence names marked with a single asterisk are from cyst nematodes (Hs and Hg), those with two asterisks are from root-knot nematodes, and the MiIDL1 peptide is highlighted in bold and underscored. The numbers under the heading of C-term SP indicate the amino acid position C-terminal from the end of the predicted signal peptide.

In an earlier study (Tucker and Yang, 2013), we concluded that MiIDL1 is more similar to the plant IDA and IDLs than CLE-like peptides. As more information has since been published regarding the phylogeny and sequence similarity among several different signaling peptides (Butenko and Simon, 2015; Stø *et al.*, 2015; Vie *et al.*, 2015) we performed a new, more extensive sequence alignment that included the active domains of these similar plant gene products: CLAVATA3/ESR-related (CLE), PAMP-INDUCED SECRETED PEPTIDE (PIP), and PIP-LIKE (PIPL) (Vie *et al.*, 2015) (Fig. 1). In addition to the IDA-like proteins from the root-knot nematodes *M. incognita*, *M. hapla*, and *M. floridensis*, we included sequences for other root-knot and cyst nematode signaling peptides that share sequence similarity with IDL, CLE, and PIP peptides (Davis *et al.*, 2008; Bobay *et al.*, 2013). Only the most conserved C-terminal motif for each of these protein classes was used in the alignment; however, we included one extra amino acid after the conserved IDA EPIP domain (Stenvik *et al.*, 2008*b*) to allow a potential alignment with the conserved domain in the PIP peptides (Fig. 1). In line with previous findings (Stø *et al.*, 2015; Vie *et al.*, 2015), we identified *IDA-like* genes in every dicot genome examined and several monocots, including banana, oil palm, and rice, and also *Pinus taedea*, a gymnosperm (results not shown). As previously observed with a much smaller set of proteins (Tucker and Yang, 2013), the root-knot nematode *IDA-like* genes clustered with the plant *IDA-like* genes and no other gene families (Fig. 1). Interestingly, the root-knot nematode 16D10 peptide, which shares similarity with IDLs and CLEs (Tucker and Yang, 2013) and is known to interact with a SCARECROW-like transcription factor (Huang *et al.*, 2006*b*), clustered with the CLE-like peptides. The clustering of Mi16D10 with CLEs demonstrates how difficult it can be to predict protein function simply based on sequence alignments. Moreover, with more sequence data available for cyst nematodes, we did not find *IDA-like* genes in any genera of cyst nematodes including the potato cyst nematode, genus *Globodera*.

### Exogenous application of synthetic MiIDL1 peptide rescues the Arabidopsis ida mutant

**Fig. 2.**
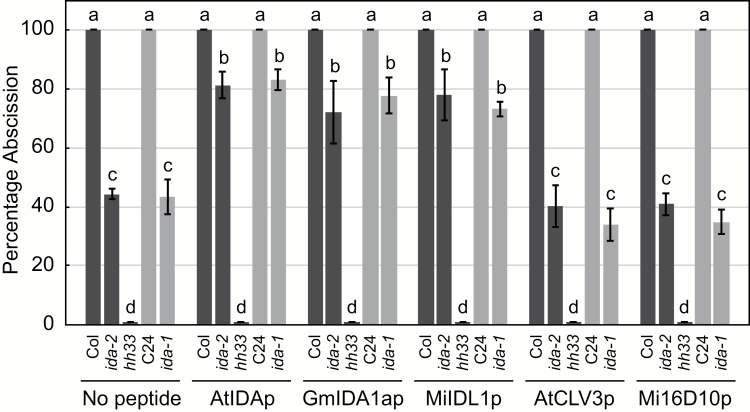
Percentage abscission of individual flowers cut from wild-type and mutant Arabidopsis plants and then treated in agar with 10 μM of the indicated peptide (listed in Table 1). Columbia (Col-0) and C24 are ecotypes of Arabidopsis that were used as controls. The mutant *ida-1* is in the C24 background (lighter gray bars), whereas, the *ida-2* and *hh33* mutants are in the Columbia background (darker gray bars). Standard error bars indicate the variance of the means for between three and five experiments and each experiment included between 10 and 30 flowers for each treatment. Letters above standard-error bars indicate means that are statistically grouped using a Tukey–Kramer test in SAS.

As a first step in determining if the nematode *MiIDL1* gene product can function as an IDA mimic, we tested whether a synthetic MiIDL1 peptide could rescue the phenotypes of the Arabidopsis *ida* mutant. The MiIDL1 peptide we used encompassed the entire translation product of the *MiIDL1* transcript starting at the end of the predicted N-terminal signal peptide (Tucker and Yang, 2013) (Table 1). For comparison, we also prepared synthetic peptides for the Arabidopsis AtIDA-EPIP (Stenvik *et al.*, 2008*b*), soybean GmIDA1a-EPIP (Tucker and Yang, 2012), root-knot nematode Mi16D10 (Huang *et al.*, 2006*b*), and the Arabidopsis AtCLV3 C-terminal motif (Olsen and Skriver, 2003) (Table 1). Each of these five peptides was used to treat *ida* mutant flowers to test for effects on petal abscission. Individual Arabidopsis flowers at anthesis were cut from wild-type (Columbia (Col-0) and C24 ecotypes) plants and the loss of function mutants *ida-1* (C24), *ida-2* (Col-0), and the *hae*-3/*hsl2-3* (*hh33*) double mutant (Col-0) and the pedicel inserted into agar in Petri plates each containing 10 μM of peptide. In line with an earlier study (Stenvik *et al.*, 2008*b*), 100% of the wild-type flower petals abscised after 48 h from both the Columbia and C24 ecotypes (Fig. 2). At 48 h, approximately 45% of the flowers abscised their petals in both the *ida-2* and *ida-1* mutants and none of the petals had abscised from the *hh33* double mutant. Treatment with 10 μM of the MiIDL1p peptide caused approximately 80% of the petals to abscise, which was similar to the percent abscission for the Arabidopsis IDA peptide (AtIDAp) and the soybean GmIDA1ap peptide (Fig. 2). However, the 10 μM treatments with the AtCLV3p peptide and Mi16D10p, which share sequence similarity with IDA and IDLs, were similar to the non-treated (no peptide) *ida* mutants, i.e. approximately 35% abscission (Fig. 2). Thus, the synthetic nematode MiIDL1p peptide rescued the abscission phenotype of *ida* mutants much like the native AtIDAp peptide and none of the treatments affected the *hh33* receptor mutant, suggesting that the MiIDL1p treatment is signaling via the same pathway as AtIDAp.

**Fig. 3.**
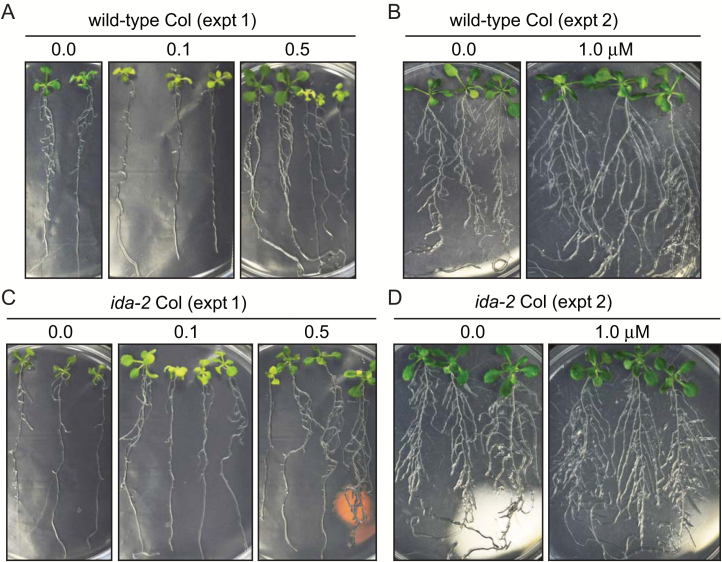
Arabidopsis wild-type Columbia (A, B) and *ida-2* mutant seeds (C, D) were germinated and grown for 2 weeks in two separate experiments on agar containing 0.0, 0.1, and 0.5 (A, C) or 0.0 and 1.0 μM MiIDL1p peptide (B, D).

We next tested the effect of MiDL1p on root development, as the Arabidopsis *ida* mutant is known to have a lower density of lateral roots, i.e. a less branched root architecture than wild-type roots (Kumpf *et al.*, 2013). We grew the wild-type and *ida* mutant seedlings on agar plates containing a series of concentrations of the synthetic MiIDL1p peptide and monitored the effect of the treatments on root development. A change in root architecture is a complex phenotype because growth of the above-ground parts (carbohydrate source tissue) affects the growth of roots (sink tissue) and, vice versa, an enhanced growth of roots (nutrient source) affects the growth of above-ground parts. Nonetheless, the *ida* mutant had a less branched root architecture (Fig. 3; Supplementary Fig. S1); moreover, when both the wild-type plants and the *ida* mutant plants were grown in the presence of different concentrations of MiIDL1p, there was a trend for the roots to be progressively more branched as the concentration of MiIDL1p increased from 0.0 to 1.0 μM (Fig. 3; Supplementary Fig. S1).

### MiIDL1 complements the *ida* mutant phenotype in transgenic Arabidopsis

**Fig. 4.**
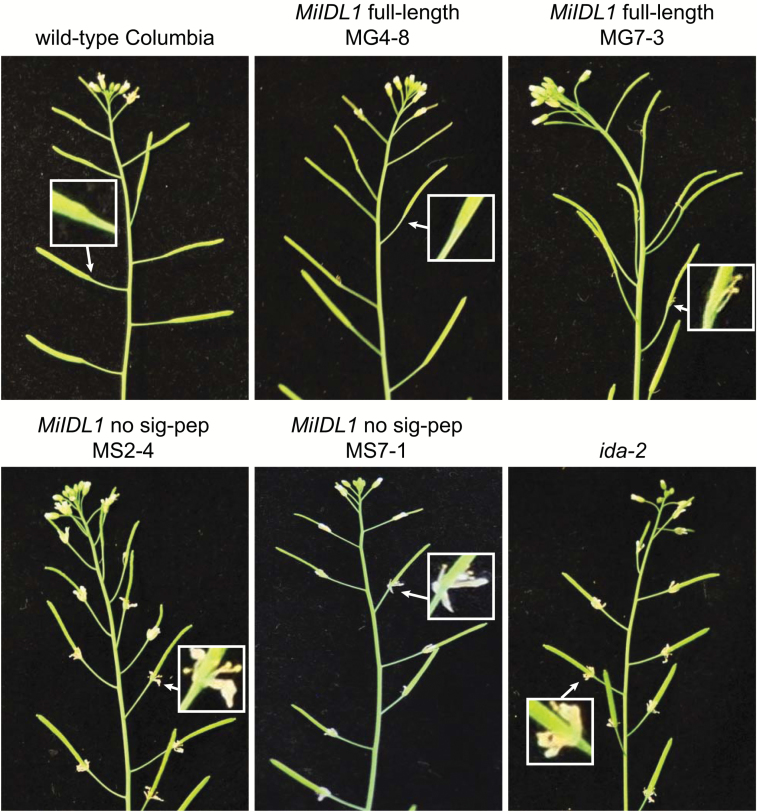
Arabidopsis *ida-2* mutant plants transformed with CaMV 35S promoter-driven full-length *MiIDL1* open reading frame (ORF), *MiIDL1* ORF minus the predicted signal peptide, or non-transformed wild-type and *ida-2* mutant plants for comparison. Two transformation events (MG4-8 and MG7-3; MS2-4 and MS7-1) are included for each of the different constructs. The boxed insets are a 4-fold zoom of the indicated silique base.

To further substantiate that *MiIDL1* can function as an IDA mimic, we transformed the *ida-2* mutant plants (Col-0) with a full-length *MiIDL1* gene that included the predicted N-terminal signal peptide (Tucker and Yang, 2013), and an *MiIDL1* construct in which the signal peptide was deleted. If a processed MiIDL1 peptide binds to the apoplast-exposed side of the HAE and/or HSL2 receptors in the plasma membrane, as would be expected for complementation of the abscission phenotype, a signal peptide would be required for translation on rough endoplasmic reticulum and secretion into the apoplast. Indeed, the transgenic *ida-2* plants that included the full-length *MiIDL1* gene (MG) displayed nearly wild-type floral organ abscission whereas the transgenic plants containing the construct lacking the signal peptide (MS) had a petal abscission phenotype very similar to the *ida-2* plants, i.e. delayed or no petal abscission (Fig. 4). Nevertheless, a range of abscission rescue was observed in transgenic plants with the full-length construct. Included in Fig. 4 are two representative transgenic lines with the full-length *MiIDL1* gene, MG4-8 and MG7-3. MG4-8 displayed a near complete recovery of wild-type abscission whereas MG7-3 displayed a partial recovery of abscission where most of the petals had abscised by position 11 but some of the stamens remained attached (Fig. 4). However, in the MS lines (minus the signal peptide) the floral organ abscission was very similar to the *ida-2* mutant, i.e. the stamens, sepals, and petals remained attached beyond flower position 11 (Fig. 4).

### RNAi suppression of the *MiIDL1* gene in Arabidopsis reduces gall numbers and size

As an initial step to determine if the *MiIDL1* gene plays a role in the infection process, we used a double-stranded, RNA interference approach (dsRNAi) to suppress expression of the *MiIDL1* gene in the nematode. It has been demonstrated that expression of a nematode-targeted RNAi construct in the host plant can inhibit expression of genes in the parasitic nematode (Dutta *et al.*, 2014). We prepared an *MiIDL1*-RNAi construct and a separate RNAi construct expressing a dsRNA for the *E. coli* β-glucuronidase (*GUS*) gene, which served as a control for these experiments. We reasoned that expression of a *GUS*-RNAi construct, which in our case would not specifically suppress the expression of any host or nematode gene but would produce a 300 bp dsRNA that would be processed into smaller RNA, would be a better control than transformation with an empty vector construct as is commonly done (Ibrahim *et al.*, 2011; Youssef *et al.*, 2013; Lischweski *et al.*, 2015). Although the most conserved 45 nt region of the *MiIDL1*-RNAi has only 28–33% identity with the Arabidopsis *IDA* and *IDLs* and is unlikely to affect their expression, we still considered the possibility that expression of the *MiIDL1*-RNAi might inhibit the native Arabidopsis *IDA*. We therefore examined flower petal abscission in the *MiIDL1*-RNAi and *GUS*-RNAi transgenics. Both *MiIDL1* and *GUS*-RNAi transgenic lines displayed a normal wild-type petal abscission phenotype and wild-type root architecture (Supplementary Fig. S2). No discernable phenotype different from the wild-type plants was observed for either the *MiIDL1* or *GUS*-RNAi plants. Thus, expression of the *MiIDL1*-RNAi did not appear to significantly suppress expression of *AtIDA* or an *AtIDL* that might have manifested itself in growth or development.

**Fig. 5.**
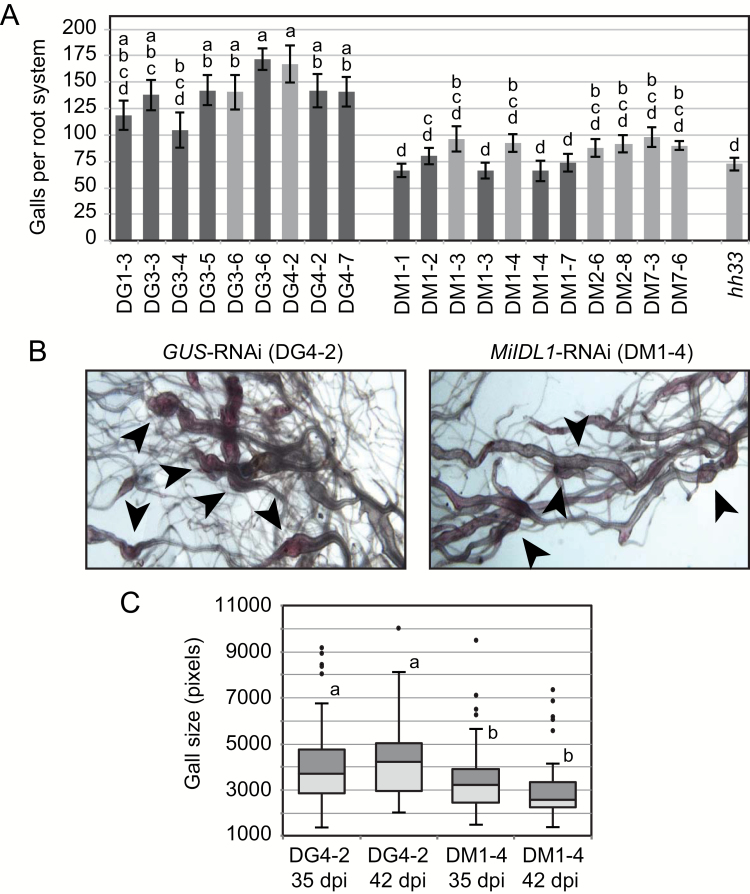
Gall number and size on *M. incognita* infected roots of wild-type Arabidopsis transformed with *GUS* and *MiIDL1*-RNAi. (A) Mean number of galls per root system of 10–12 independently grown plants for multiple transgenic lines for *GUS* (DG) and *MiIDL1* (DM) at 35 dpi (light gray bars) and 42 dpi (darker gray bars). (B) Photos showing the relative size of galls at 35 dpi on *GUS* and *MiIDL1*-RNAi plants. (C) Box chart of gall sizes (pixels counted using ImageJ software) from multiple photos of different plants from the lines indicated (the area of 50–100 galls was measured per transgenic line). In (A), DG and DM indicate *GUS* or *MiIDL1*-RNAi, respectively, and the first number after DG or DM indicate the transformation event from T0 plants and the second number the seed collection (line) from T1 plants. *hh33* is the *hae-3*/*hsl2-3* double mutant. Galls were counted on T3 plants. The standard error bars in (A) reflect gall counts for 9–14 plants for each line. Letters above or next to the bars in (A, C) indicate means that are statistically grouped using a Tukey–Kramer test in SAS. The *P* value (two-tailed *t*-test) for the significance of the difference between the means for the *GUS* lines compared with the *MiDL1* lines in (A) is 4.1 × 10^−7^. Arrows in (B) point to representative galls. Points in (C) are outliers.

Multiple transgenic plants for both *MiIDL1*-RNAi and *GUS*-RNAi were infected with *M. incognita* and the roots examined at 2, 3, 7, 14, 15, 35, 42, and 56 d post-inoculation (dpi). To quantify the number of galls per root system, infected roots were harvested at 14, 35, and 42 dpi and stained with acid fuchsin to enhance detection of the nematodes (Bybd *et al.*, 1983). At 14 dpi, when the galls were just beginning to form and be detectable under our conditions, we did not observe any clear reduction in gall number. At 35 and 42 dpi there were approximately 40% fewer galls on the *MiIDL1*-RNAi roots relative to *GUS*-RNAi roots (Fig. 5A). In addition, the galls that formed on the *MiIDL1*-RNAi plants appeared smaller than the galls on the *GUS*-RNAi plants (Fig. 5B, C).

**Fig. 6.**
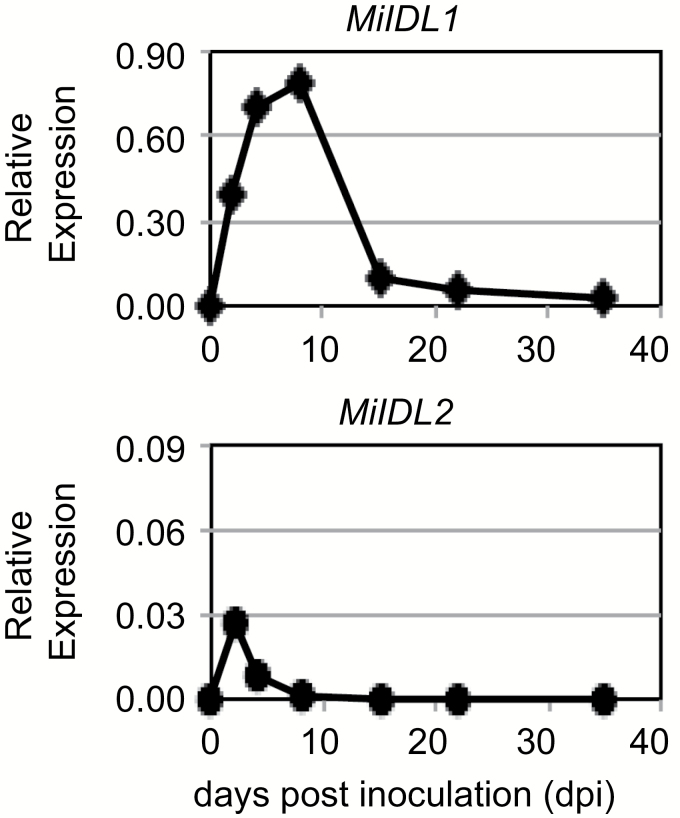
Gene expression profile for *MiIDL1* (diamonds) and *MiIDL2* (circles) normalized to the nematode elongation factor 1b (*MiEF1b*) in wild-type Arabidopsis infected with root-knot nematodes (*M. incognita*).

There are two *IDA-like* genes in *M. incognita*, *MiIDL1* and *MiIDL2* (Tucker and Yang, 2013). *MiIDL1* and *MiIDL2* are both expressed early after infection of wild-type Arabidopsis; however, *MiIDL2* abundance is 10-fold less than *MiIDL1* (Fig. 6). *MiIDL2* is 90% identical to *MiIDL1* and it is likely that the *MiIDL1*-RNAi would suppress both *MiIDL1* and *MiIDL2*. Our focus for these early experiments was to establish a significant change in infection or gall development on *MiIDL1*-RNAi plants; nonetheless, total RNA was isolated from a sampling of *MiIDL1* and *GUS*-RNAi-infected roots at 14 and 42 dpi. All the transgenic plants had high levels of the transcript for *NPTII* (the selection marker) and the *GUS* or *MiDL1* transcripts in the respective transformed plants (Supplementary Fig. S3). We did not, however, see a significant reduction of *MiIDL1* expression in the *MiIDL1*-RNAi lines at either 14 or 42 dpi; nonetheless, we did see an approximately 40% reduction in the constitutively expressed nematode elongation factor, *MiEF1b*, at 42 dpi, which supports the reduction in gall numbers that we observed at 42 dpi (Supplementary Fig. S3).

## Discussion

INFLORESCENCE DEFICIENT IN ABSCISSION (IDA) is highly conserved in plants. IDA and IDA-like peptides, however, have sequence similarity with CLE and PIP and PIP-like peptides but each has functionally distinct activities in the plant (Tucker and Yang, 2012; Vie *et al.*, 2015). Moreover, although each of the plant IDA, CLE, and PIP peptides activates LRR-RLKs within the same class of RLKs (Class XI), they each bind to different RLKs (Hou *et al.*, 2014; Tabata *et al.*, 2014). The MiIDL1 peptide is more similar to plant IDA-like peptides than CLE or PIP peptides (Fig. 1), but sequence similarity over a short stretch of amino acids is not sufficient to prove that the peptide can function as an IDA-like signal. An example of this would be Mi16D10, which has sequence similarity with CLE peptides (Fig. 1) but binds to and alters the activity of a SCARECROW-like transcription factor (Huang *et al.*, 2006*b*).

We have demonstrated herein that indeed the root-knot nematode *MiIDL1* gene product can function as an IDA signal. Treatment of excised Arabidopsis *ida* mutant flowers with the nematode MiIDL1p peptide rescued the delayed abscission phenotype, as did the AtIDAp and GmIDA1ap peptides, whereas the AtCLV3p and Mi16D10p peptides did not rescue the *ida* mutant phenotype (Fig. 2). Moreover, transformation of the Arabidopsis *ida-2* mutant with full-length and signal-peptide-attenuated MiIDL1 constructs indicates that not only can the MiIDL1 peptide function as an IDA signal but it also must be secreted into the apoplast to be functional (Fig. 4). Thus, the reversal of the *ida* mutant phenotype by MiIDL1 indicates that the *MiIDL1* gene produces a protein that presumably can bind to the HAE/HSL2 receptors to propagate the signaling events necessary to complement the mutant phenotype. Interestingly, however, we did not observe an accumulation of a white substance reportedly rich in arabinogalactan at the abscission zone (AZ) in the MiIDL1-overexpressing transformants as was observed for the overexpression of the Arabidopsis *IDA* and *IDLs* in Arabidopsis (Stenvik *et al.*, 2006, 2008*b*). The lack of a white secretion may be unique to MiIDL1 or simply reflects nuances in how IDA and IDA-like signals elicit a signaling pathway in Arabidopsis.

The Arabidopsis IDA precursor protein is secreted into the apoplast where it is processed into a smaller peptide (Stenvik *et al.*, 2008*b*; Butenko and Simon, 2015; Schardon *et al.*, 2016). The secreted IDA protein is predicted to be cleaved by subtilisin-like serine proteinases at both the N-terminus and C-terminus resulting in a 14 amino acid bioactive product (GVPIPPSAPSKRHN) (Schardon *et al.*, 2016; Stührwohldt *et al.*, 2017). When the N-terminal cleavage site was blocked with N-methylation of the Lys/Gly bond in a longer 23 amino acid peptide (SHIFGYLPK[m]GVPIPPSAPSKRHN), the methylated peptide was completely inactive whereas the non-methylated 23 amino acid peptide retained activity. This is interesting in regard to the prediction of the end of the signal peptide for MiIDL1. Previously, we predicted the most probable N-terminal cleavage site of the MiIDL1 for the signal peptide between amino acids 25 and 26 (Ser and Ile) (Tucker and Yang, 2013); however, a new version of SignalP (version 4.1) (Petersen *et al.*, 2011) predicts two possible signal peptide cleavage sites, one at the Ser/Ile and another with slightly higher probability three amino acids further down between the Gly and Val (Table 1), which would remove the glycine at the N-terminus of the predicted bioactive IDA peptide (Schardon *et al.*, 2016). The earlier Ser/Ile signal peptide cleavage site for MiIDL1 (Tucker and Yang, 2013) was used to prepare the MiIDL1p peptide (Table 1) that was used for rescue of the *ida* phenotype (Fig. 2). The MiIDL1p peptide clearly rescued the *ida* phenotype; however, we do not know if this peptide is proteolytically cleaved in the apoplast at the N-terminus or C-terminus to produce a shorter peptide *in vivo*. Also of interest is that, when the proline within the highly conserved active domain S(A/G)PS is hydroxylated, the peptide binds much more strongly to the HAE and HSL2 receptors (Butenko *et al.*, 2014). The MiIDL1p peptide did not contain a hydroxyproline and typically it would be assumed that an exogenous application of a peptide would not be hydroxylated *in vivo* since proline hydroxylation normally occurs in the endoplasmic reticulum prior to secretion (Yuasa *et al.*, 2005; Vlad *et al.*, 2007). In addition, others have demonstrated that the *ida* mutant phenotype could be rescued with IDA-like peptides that do not contain a hydroxyproline modification (Stenvik *et al.*, 2008*b*; Schardon *et al.*, 2016). Interestingly, a recent report argues that the exogenously applied peptides might still be hydroxylated at proline by an extracellular enzyme (Stührwohldt *et al.*, 2017).

To determine the importance of *MiIDL1* expression to *M. incognita* pathogenesis, we transformed wild-type Arabidopsis with a 324 double-stranded RNAi construct of *MiIDL1* that we predicted would inhibit *MiIDL1* expression in the nematode (Dutta *et al.*, 2014). There are, however, two *IDA-like* genes in *M. incognita*, *MiIDL1* and *MiIDL2*; however, *MiIDL2* is expressed at a much lower level, 10-fold less, than *MiIDL1* (Fig. 6). Nevertheless, *MiIDL2* is 90% identical to *MiIDL1* and it is expected that the *MiIDL1*-RNAi would suppress both *MiIDL1* and *MiIDL2*. In regard to phenotypes associated with the expression of the *MiIDL1*-RNAi, we did not observe a reduction in gall number at 14 dpi when galls were just beginning to form, but we did observe a significant 40% reduction in gall numbers at 35 and 42 dpi as well as notably smaller galls at these time points (Fig. 5). RNAi inhibition of the nematode gene requires ingestion by the nematode of the processed ~22 bp RNAi (Huang *et al.*, 2006*a*). *Meloidogyne incognita* does not feed until it has established a rudimentary feeding structure (giant cells), which can be several days post-inoculation (Wyss and Grundler, 1992), and feeding stops when the J2 progressively molts into an adult female (Jung and Wyss, 1999). At 14 dpi *MiIDL1* expression is already declining relative to a constitutively expressed *MiEF1b* gene but is near the peak of expression per nematode (i.e. normalized to expression of an Arabidopsis actin gene *AtACT2*) (Supplementary Fig. S3). Under our soil infection conditions, we did not see a significant reduction of *MiIDL1* expression in the *MiIDL1*-RNAi lines at either 14 or 42 dpi, but did observe approximately 40% reduction in the constitutively expressed nematode elongation factor, *MiEF1b*, at 42 dpi, which reflects the 40% reduction in gall numbers on the *MiIDL1*-RNAi roots at 42 dpi (Supplementary Fig. S3). Because we did not observe any plant phenotype associated with the *MiIDL1*-RNAi or *GUS*-RNAi lines relative to wild-type Arabidopsis, it is reasonable to conclude that the *MiIDL1*-RNAi reduction in gall numbers is due to a suppression of the native *MiIDL1/2* genes. The *MiIDL* mRNAs are low-abundance transcripts in the root RNA, which makes it difficult to quantify small changes in expression, and, moreover, the peak of expression of *MiIDL1* may shift due to a change in the development of the nematode feeding structure as a result of the RNAi inhibition of *MiIDL1*; nonetheless, an extensive time course collection of RNA with more experimental replication may confirm a suppression of mRNA accumulation.

Our results indicated that gall numbers on *hh33* roots were very similar to those observed for *MiIDL1*-RNAi roots, which was approximately 40% fewer than on wild-type Arabidopsis roots (Fig. 6A). This observation in the context of the complementation results supports a model where MiIDL1 functions through binding and activation of HAE, HSL2, or a similar HASEA-like receptor in the Arabidopsis roots. However, although our results strongly suggest that MiIDL1 is secreted from the nematode and is deposited directly into the apoplast or is somehow translocated to the apoplast, this remains to be confirmed experimentally.

As mentioned above, IDA was demonstrated to play an important role in Arabidopsis floral organ abscission and lateral root emergence and presumably the regulation of genes associated with cell wall modification that accompanies these developmental processes (Butenko *et al.*, 2003; Kumpf *et al.*, 2013). An up-regulation of gene expression for cell wall modifying *EXPs* and *PGs* is well established for root-knot nematode infection of Arabidopsis (Jammes *et al.*, 2005; Cabrera *et al.*, 2014). Thus, our original hypothesis for the role of the MiIDL1 peptide (Tucker and Yang, 2013) was that it might play a role in cell wall modifications associated with formation of the gall similar to that described for abscission and lateral root emergence (Butenko *et al.*, 2003; Kumpf *et al.*, 2013). However, recently it was reported that *AtIDL6* and *AtIDL7* play a role in suppressing defense and stress responses in the plant (Vie et al., 2015, 2017; Wang *et al.*, 2017). *AtIDL6* and *AtIDL7* are rapidly up-regulated by PAMPs, e.g. flg22, and *Pseudomonas syringae* pv. tomato (Pst) DC3000 (Vie *et al.*, 2015, 2017; Wang *et al.*, 2017). In the particular case of *P. syringae*, up-regulation of *AtIDL6* increased the number of bacterial infections (Wang *et al.*, 2017). One explanation given for the enhanced infection was that the AtIDL6 peptide acted through HAE/HSL2 to up-regulate genes linked to cell wall modification, which included the polygalacturonase *AtADPG2* (*AtPGAZAT*). They proposed that expression of these enzymes loosens the cell wall and aids the assembly of a type III secretion system that the bacteria use to inject virulence factors. Also of interest, Wang *et al.* (2017) suggested that expression of *AtIDL6* directly or indirectly suppressed a salicylic acid (SA)-dependent defense response, which was indicated by an *AtIDL6*-dependent decrease in *PATHOGENESIS-RELATED PROTEIN 1* (*PR1*) expression. A more recent study demonstrated that AtIDL7 and possibly AtIDL6 peptides were negative regulators of stress-induced ROS-signaling (Vie *et al.*, 2017). Treatment of Arabidopsis leaves with either AtIDL6 or AtIDL7 peptide caused a significant decrease in several stress-related genes including *ZINC FINGER PROTEIN* (*ZFP*), *WRKY*, and genes encoding calcium-dependent proteins (Vie *et al.*, 2017).

Suppression of the SA- and ROS-associated defense response or stress are interesting in regard to a potential role for MiIDL1 as elevation of an SA and ROS defense response has been implicated in root-knot nematode resistance (Branch *et al.*, 2004; Goverse and Smant, 2014), and nematode migration and infection of roots elevates many defense- and stress-associated genes (Gheysen and Fenoll, 2002; Jammes *et al.*, 2005; Alkharouf *et al.*, 2006; Puthoff *et al.*, 2007). Recent studies to characterize nematode elicitors have identified several that are involved in suppressing host defense and stress responses (Goverse and Smant, 2014; Hewezi, 2015). Moreover, it has been demonstrated that an extracellular network of LRR-RKs, which includes the HAESA-like receptors, is utilized to balance the response to extracellular signal (Smakowska-Luzan *et al.*, 2018). Determining the mechanism of action for the IDA-like effectors in root-knot nematode infection awaits further research. Our original hypothesis was that MiIDL expression would directly affect gall development but it seems equally possible that MiIDL expression is involved in moderating stress and defense responses in the host; nonetheless, our findings here are significant in that we demonstrate that MiIDL1 can mimic IDA in Arabidopsis and that the MiIDL1 protein likely signals through HAE, HSL2, or another HAESA-like receptor.

## Supplementary data

Supplementary data are available at *JXB* online.

Fig. S1. Arabidopsis wild-type C24 and *ida-1* mutant seeds were germinated and grown for 2 weeks in two separate experiments on agar containing 0.0, 0.1, and 0.5 or 0.0 and 1.0 μM MiIDL1p peptide.

Fig. S2. Arabidopsis seedlings of wild-type (Columbia) plants transformed independently with the *GUS*-RNAi and *MiIDL1*-RNAi constructs.

Fig. S3. Mean relative expression days post inoculation (dpi) of T-DNA and native *MiIDL1* in *GUS* and *MiIDL1*-RNAi events and lines as included in Fig. 5A, and time course expression of *MiIDL1* in inoculated wild-type Arabidopsis.

Table S1. Accession numbers or sequence for gene motifs included in Fig. 1.

Table S2. Primers for qPCR and construct preparation.

Supplementary Figures S1-S3

Supplementary Table S1

Supplementary Table S2

## Funding

This work was supported by a US-Israel Binational Agricultural and Development Fund (BARD) US-4571-12C grant to MLT, Shimon Meir and Sonia Philosoph-Hadas, the United States Department of Agriculture (USDA Project 1245-21220-232-00D) and by the National Research Foundation of Korea (NRF) grant funded by the Korean government (Ministry of Science, ICT & Future Planning) (NRF-2017R1A2B4010356) to JK.
