# Supplementary material for: The root-knot nematode Meloidogyne incognita produces a functional mimic of the Arabidopsis INFLORESCENCE DEFICIENT IN ABSCISSION signaling peptide
Source: J Exp Bot. 2018 Apr 10;69(12):3009–21. doi: 10.1093/jxb/ery135 (PMC5972575; doi:10.1093/jxb/ery135)
Supplement: Supplementary Figures S1-S3 [file ery135_suppl_supplementary-figures_s1-s3.pdf]

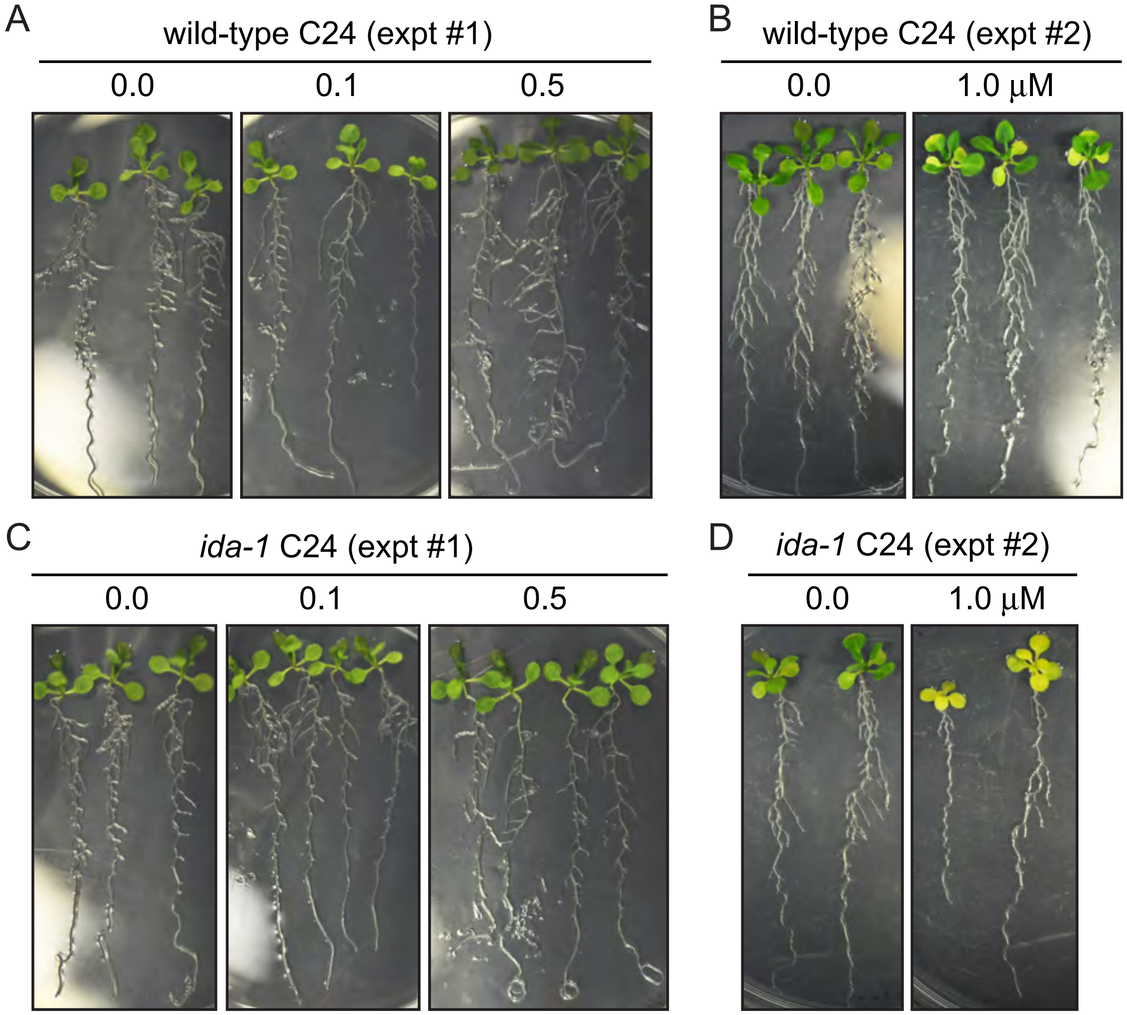

Supplementary Fig. S1. Arabidopsis wild-type C24 (A and B) and *ida-1* mutant seeds (C and D) were germinated and grown for 2 weeks in two separate experiments on agar containing 0.0, 0.1, and 0.5 (A and C) or 0.0 and 1.0 μM MiIDL1p peptide (B and D).

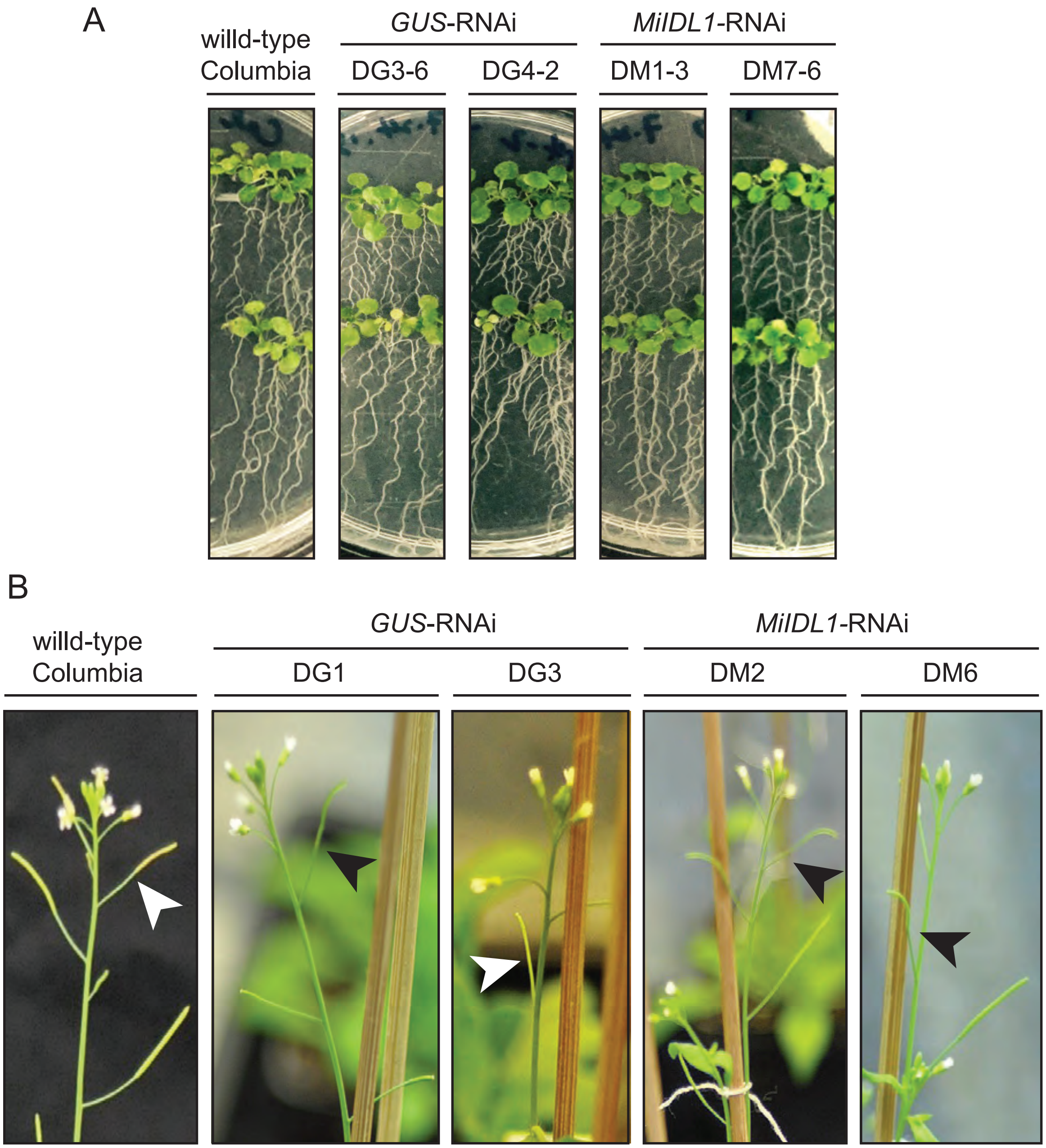

Supplementary Fig. S2. Arabidopsis seedlings of wild-type (Columbia) plants transformed independently with the *GUS*-RNAi and *MiIDL1*-RNAi constructs. (A) Seedlings germinated on agar displaying root phenotype. (B) Seedlings grown in soil displaying floral organ abscission phenotype. The arrows in (B) indicate the first silique inwhich all the petals, stamens and sepals have fully abscised.

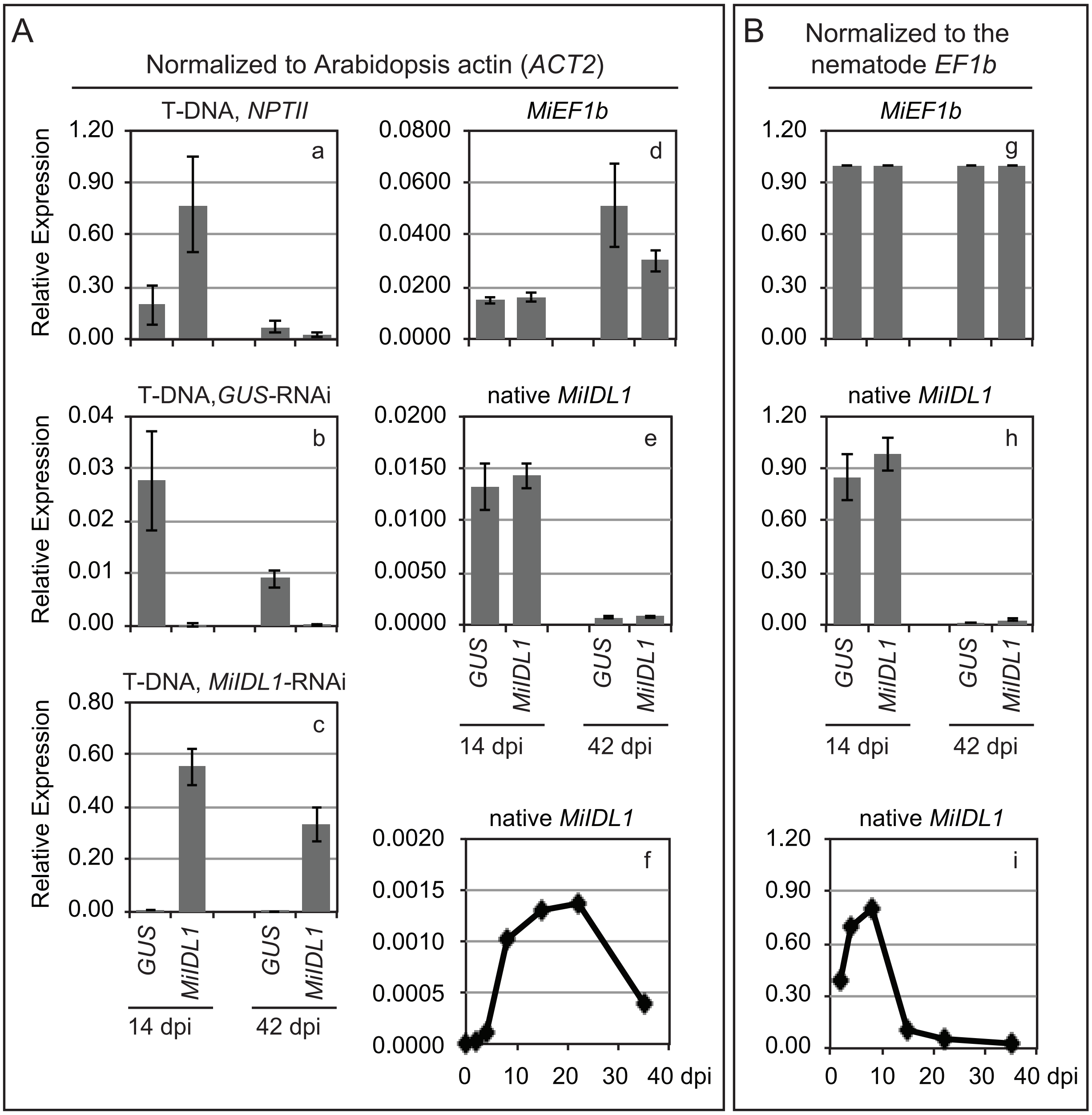

Supplementary Fig. S3. Mean relative expression days post inoculation (dpi) of T-DNA and native *MiIDL1* in *GUS* and *MiIDL1*-RNAi events and lines as included in Figure 5A, and time course expression of MiIDL1 in inoculated wild-type Arabidopsis. (A) Gene expression normalized to Arabidopsis actin *AtACT2* or (B) expression normalized to the nematode *ELONGATION FACTOR 1b* (*MiEF1b*). Graphs a, b and c, expression of genes in the T-DNA inserts; graphs d-i, expression of the native nematode genes MiEF1b and MiIDL1 in transgenic RNAi plant (d, e, g and h) or inoculated wild-type Arabidopsis (f and i).
