# Supplementary material for: The root-knot nematode Meloidogyne incognita produces a functional mimic of the Arabidopsis INFLORESCENCE DEFICIENT IN ABSCISSION signaling peptide
Source: J Exp Bot. 2018 Apr 10;69(12):3009–21. doi: 10.1093/jxb/ery135 (PMC5972575; doi:10.1093/jxb/ery135)
Supplement: Supplementary Table S1 [file ery135_suppl_supplementary-table_s1.pdf]

Supplementary Table S1. Accession numbers or nucleotide and protein sequence for the gene motifs used in the dendrogram in Figure 1. Sequences without an accession number were identified by us in genomic and EST sequences and tested for expression by qPCR (Tucker and Yang, 2012, 2013).

Name in Fig. 1	Nucleotide Accession	Protein Accession	Other
AtCLE41	NM_113389.1	NP_566754.1	AT3G24770
AtCLV3	NM_001124926.1	NP_001118398	AT2G27250.3
AtIDA	NM_105550.1	NP_564941	At1G68765
AtIDL1	NM_113464.2	NP_566774.1	At3G25655
AtIDL2	NM_001085327.2	NP_001078796.1	At5G64667
AtIDL3	NM_001085091.1	NP_001078560.1	At5G09805
AtIDL4	NM_001084711.1	NP_001078180.1	At3G18715
AtIDL5	NM_001084366.2	NP_001077835.2	At1G76952
AtIDL6	NM_001342808.1	NP_001318482.1	At5G05300
AtIDL7	AC009991.5	AAF01528.1	At3G10930
AtIDL8	AK221754.1	BAD93814.1	
AtPIP1	NM_118988.3	NP_194575.2	At4G28460
AtPIP2	NM_119892.2	NP_568022.1	At4G37290
AtPIP3	NM_127891.1	NP_565549.1	At2G23270
AtPIPL2	NM_111484.2	NP_187260.1	At3G06090
AtPIPL3	NM_119893.2	NP_568023.1	At4G37295
AtPIPL4	NM_001125892.1	NP_001119364.1	At5G43066
AtPIPL5	NM_001344469.1	NP_001331732.1	At5G43068
GmCLV3a	XM_006594862.2	XP_006594925.1	Glyma.13G313400.1
GmIDA1a	appended below	appended below	Glyma.10G205400.1
GmIDA2a	appended below	appended below	Glyma.01G090400.1
GmIDA3a	appended below	appended below	Glyma.04G215100.1
GmIDA4a	appended below	appended below	Glyma.05G189500.1
GmIDA5a	appended below	appended below	Glyma.20G165500.1
GmIDA6a	appended below	appended below	Glyma.16G170400.1
HgCLE1	FJ503004.1	ACT32609.1	
HgCLE2	FJ503005.1	ACT32610.1	
HsCLE1	HM588679.1	AEA06591.1	
HsCLE2	HM588680.1	AEA06592.1	
HsCLEB	KY124382.1	AQR58088.1	
MfIDL1	appended below	appended below	
MhIDL1	appended below	appended below	
Mi16D10	AY134435.1	AAN08582.1	
MiIDL1	KC237722.1	AGF86396.1	
MiIDL2	appended below	appended below	
MiMAP1-V1	AJ278663.1	CAC27774.1	
MiMAP1-V4	AJ278663.1	CAC27774.1	
MiMAP1-V5	AJ278663.1	CAC27774.1	
MiMAP1-V7	AJ278663.1	CAC27774.1	
SlCLV3	XM_010315155.2	XP_010313457.1	Solyc11g071380.1.1
SlIDA1	appended below	appended below	Solyc05g010000.1.1

SlIDA2	appended below	appended below	
SlIDA3	appended below	appended below	
SlIDA4	appended below	appended below	Solyc07g044890.1.1
SlIDA5	appended below	appended below	

Sequences which do not have an accession number at NCBI.

Nucleotide:

```
>GmIDA1a ORF
ATGGCCAATTCTCATTATTCTAAAACTTTGCATCTTCCATGCAAATCCTTCTCTATGGCT
ATTCTCCTTGTTTACTTGCTTCTTGTTGGTTCTTGCACTGCAATAAGGACAGGCGCAACC
ATGCGGTTGAATGAAGGCTCAGAACTTCTCCGGCGGAAGCAGCAACAACCGCGATTTCCT
TACAAAGGTCTTGTTTTCAACTTCTTACCCAAAGGGGTGCCAATTCCACCTTCAGGCCCT
TCAAAGAGGCACAACTCAGTGGTGGCTTCCACACCTAAGAATTGA
>GmIDA2a ORF
ATGGCAAATTACCATTCTTCTAAATCCTTGCATCTTCCATGCAAAACCTTATCTCTAGTT
CTCCTTTTTGTTTACTTGCTACTTATTGGTTCTTGTGATGCAATAAGAATAGGCCAAACT
ATGAAGCTGAATGAAACAAGAGAGATTCTCAAGAGAAAGCGCAATAACCAACATGGTTTT
CCATACAAAAGCATGGTTTTCAACTTCTTCCCCAAAGGGCCGGTGCCACCTTCAGGTCCT
TCAAAGAGGCATAACGCAGTGGTGGATTCAACACCTAATAATTGA
>GmIDA3a ORF
ATGGTAGCTCTACGTAGTAGGAGACGTAAAACTCTAATGCTCTTGCTGGTCTTGTTTCTC
TGCATATTGGGGCACGGTCATGGATCAAGAACCACCAACGTCTTCAAATTGAAGCCAAAG
TCTCAGCACACGGGTCACTTTTTTGGATTCTTGCCAAAGAGGATACACATACCGTTCTCC
TCTCCTTCAAAGAAACACAATGACATTGGCCTACAAAGTTGGAGATCACCCTAA
>GmIDA4a ORF
ATGGTTTTGCATAGGAGACCTCTGATTCTAGTAATCATCTGGTTGTTCTTGTTTCTCTTC
ATATTGGGTCACTGCCATGGTTCTAGAGCCACAACAACCGTATTCAAATTCAAACCCAAA
TCTCCACATCATGGTCACTTCTCTGGCTTCTTGCCCAAAAGGATGCCTATACCGTATTCA
ACTCCTTCAAGGAAACACAATGACATTGGCTTAAGAAGTTGGAGATCACCCTGA
>GmIDA5a ORF
ATGGATAGAAGATACCTCAACTTGGTTCCAGTGCTGATGCTTTTGGTAATATGTTTTGTT
GGCCATAGTTATGGTGCTAGACACTCTAATCAAGTTTTCAAAGTGCAGCCCAGGGGAGAG
GCTTTCCCTCCAAGCTTCTTTGGTTTCTTGCCAAAAGCAATGCCAATACCACCTTCAGGG
CCTTCAAGGAAACACAACGGCATTGGATTACAAAGCTCAATGGGGGAACCATAG
>GmIDA6a ORF
ATGGGTAGAAGACATCTTCATGTATTGCCAATGCTGTTTTTGCTGATTATATACTTGGTT
GGTGCTAGTTATGGCTCTAGACACACTCAAGTGTTCAATATTCAGCCCAAATCTCAAGGT
TTACATCAAAATTTCTTTGGCTTCTTGCCAAAGGCAATGCCATTGCCACCTTCAGGGCCT
TCTAAGCAGCACAACAGCATTCAATTACAAAACTCAAGGGGGAAACAACCATAG
>SlIDA1 ORF
ATGGCTTTCTCTTTTTCTTCTTCAAAAACCCTTTATTTATCAAGCAAATTAACTTGTTTG
ATACTTGTTATTTCTCTACTTTTTAATTATGGTCATATTGTTGAAGCATCAAGATTTGGG
AGAATTATGATGGTAGAGGAAAATTCAAGAATATTTTCATCACAACATATGAAGGTATAC
AAAAAAGAAAATGCATACAAAGTTGATAATTTATTATTTACTATGTTACCAAAAGGGATT
CCAATTCCTCCTTCTGCTCCATCAAAGAGACATAATGCTATTGAAGACTCTACACCTCAA
AATTGA
>SlIDA2 ORF
ATGTTGAAAAAAAAATCACAACACAACACTATTAATTTATTTACTTCTTGTGATTTTGGTG
GTTGATCATCATGATCACCATGTTAATGCAGTAAAAAACTCACAAGTTGTTAATGTTAAG
```

CCCTTATTACCTAGTAATAACAATTCTAAATCATCTTTTTCTCAATCTTTGCCAAAAGGA
GTCCCTATTCCACCTTCTGCTCCTTCCAAAAGGCACAATCAAATCAATATATGA
>SlIDA3 ORF
ATGGAAAAAATGAGCATAAAAAACACAACTACAATATCAATCATTTTTGTCCTTGTGATA
ATAATTCAACATGCTCATGGTGCAAGTCACACACAATTTTTCAAGGTGAAGCCTTTGCCA
ATTAGTAATAAAAACAATAAATCTCCTTATTATGAGTCTTTGCCAAAAGGAGTCCCAATT
CCACCTTCTTCTCCTTCAAAAAGACACAATGGAATCAACCTCAAAAGGTATTGGCCATGA
>SlIDA4 ORF
ATGGCTTATTCTGCTAATTCCAAAACCCTTCATTATATTTCCTCATGGAAATTCATATGC
TTGATTCTCACACTATCTCTTGTTCTTGACCATGGACATGGTACTACATGCCCACCTACG
CCGTCACGTATGCCGAGGCGTTTGAAAGAGGAAGCTTCTAGAATGTTCTCAGAACTTTCT
GATGAAAAGAAAGAGTTCCTCAGCAGTACTAGTAACCGGTTCCATATGCTACCAAAAGGG
ATTCCTATTCCTCCTTCTGCACCATCAAAAAGGTGCAATTAA
>SlIDA5 ORF
ATGGCTTATTCTACTACTAATTATTCTTCATGGAAATTCATGTACTTAATTCTCACATTA
TCTCTTGTTCTTGGCTATGCTTCTTCAGTGAGAATATCGTCGACGATGAATTCAAAAGAT
GAAGACGCTTATACACTATTTTCAGAACCATCTCCAAAATATGAAGACGCTTATACACTA
TTTTCAGAATCATCTCCGAGATATTACGATGAAAATGAAAAGAAAGAGTTTCAAAAGAGT
AATTTGTTTCATATGCTACCAAAAGGGACTCTAATTCCTCCTTCTGGACCATCACGTAGA
CACAATGGAGATGGTGACTTATCTAATTATCCTTAA
>MfIDL1 ORF
ATGTTTTATTCAATTAAAAATTTAATTAATTTAATTATTATTTTAATGCTTTTATCTGTC
TCAATTGTGGATTCAATTAAAGGAGTTCCACCTAATAGTGGACCATCACACAAAGGAAAT
AAAGTTCCAGGTCCCGGCCGATAG
>MhIDL1 ORF
ATGTTTTTTTCAATTAAAAATTTGTTTAAAATTTTAATTCCTCTGATTATTTTAATGTTT
TTGAATGTTTCTTCTGTTGTTGCTCCTAAAAACATTCCTGTTCCTGCTAGTGGACCATCA
AAGAGACACAATGGAAATGGTCAAGATATAAGTAATTGA
>MiIDL1 ORF
ATGTTTTATTCAATTAAAAATTTAATTAATTTAATTATTATTTTAATGCTTTTATCTGTC
TCAATTGTGGATTCAATTAAAGGAGTTCCACCTAATAGTGGACCATCACGCAGAGGAAAT
AAAGTTCCAGGTCCCGGCCGATAG
>MiIDL2 ORF
ATGTTTTATTCATTTAAAAATTTAATTAATTTAATTATTATTTTAATGCTTTTATCTGTC
TCAATTGTGGATGCAGTTAAAGGAGTTCCACATAATAGTGCACCATCACGAAGAGGAAAT
AAATTCCCTCCAGGTCATCCAGGCTGA

Protein:

>GmIDA1a
MANSHYSKTLHLPCKSFSMAILLVYLLLVGSCTAIRTGATMRLNEGSELLRRKQQQPRFP
YKGLVFNFLPKGVPIPPSGPSKRHNSVVASTPKN
>GmIDA2a
MANYHSSKSLHLPCKTLSLVLLFVYLLLIGSCDAIRIGQTMKLNETREILKRKRNNQHGF
PYKSMVFNFFPKGPVPPSGPSKRHNAVVDSTPNN
>GmIDA3a
MVALRSRRRKTLMLLLVLFLCILGHGHGSRTTNVFKLKPKSQHTGHFFGFLPKRIHIPFS
SPSKKHNDIGLQSWRSP
>GmIDA4a
MVLHRRPLILVIIWLFLFLFILGHCHGSRATTTVFKFKPKSPHHGHFSGFLPKRMPIPYS
TPSRKHNDIGLRSWRSP
>GmIDA5a
MDRRYLNLVPVLMLLVICFVGHSYGARHSNQVFKVQPRGEAFPPSFFGFLPKAMPIPPSG

```
PSRKHNGIGLQSSMGEP
>GmIDA6a
MGRRHLHVLPMLFLLIIYLVGASYGSRHTQVFNIQPKSQGLHQNFFGFLPKAMPLPPSGP
SKQHNSIQLQNSRGKQP
>SlIDA1
MAFSFSSSKTLYLSSKLTCLILVISLLFNYGHIVEASRFGRIMMVEENSRIFSSQHMKVY
KKENAYKVDNLLFTMLPKGIPIPPSAPSKRHNAIEDSTPQN
>SlIDA2
MLKKNHNTTLLIYLLLVILVVDHHDHHVNAVKNSQVVNVKPLLPSNNNSKSSFSQSLPKG
VPIPPSAPSKRHNQINI
>SlIDA3
MEKMSIKNTTTISIIFVLVIIIQHAHGASHTQFFKVKPLPISNKNNKSPYYESLPKGVPI
PPSSPSKRHNGINLKRYWP
>SlIDA4
MAYSANSKTLHYISSWKFICLILTLSLVLDHGHGTTCPPTPSRMPRRLKEEASRMFSELS
DEKKEFLSSTSNRFHMLPKGIPIPPSAPSKRCN
>SlIDA5
MAYSTTNYSSWKFMYLILTLSLVLGYASSVRISSTMNSKDEDAYTLFSEPSPKYEDAYTL
FSESSPRYYDENEKKEFQKSNLFHMLPKGTLIPPSGPSRRHNGDGDLSNYP
>MfIDL1
MFYSIKNLINLIIILMLLSVSIVDSIKGVPPNSGPSHKGNKVPGPGR
>MhIDL1
MFFSIKNLFKILIPLIILMFLNVSSVVAPKNIPVPASGPSKRHNGNGQDISN
>MiIDL1
MFYSIKNLINLIIILMLLSVSIVDSIKGVPPNSGPSRRGNKVPGPGR
>MiIDL2
MFYSFKNLINLIIILMLLSVSIVDAVKGVPHNSAPSRRGNKFPPGHPG
```
