# Supplementary material for: The root-knot nematode Meloidogyne incognita produces a functional mimic of the Arabidopsis INFLORESCENCE DEFICIENT IN ABSCISSION signaling peptide
Source: J Exp Bot. 2018 Apr 10;69(12):3009–21. doi: 10.1093/jxb/ery135 (PMC5972575; doi:10.1093/jxb/ery135)
Supplement: Supplementary Table S2 [file ery135_suppl_supplementary-table_s2.pdf]

Supplementary Table S2. Primers for qPCR and construct preparation.

Name	Sequence	Description
AtACT2-F	TCCCTCAGCACATTCCAGCAGAT	AtACT2 constitutive expression for qPCR
AtACT2-R	AACGATTCCTGGACCTGCCTCATC	AtACT2 constitutive expression for qPCR
MiEF1b-f1	GGAAGTGATGAATCCGATGATGAGG	MiEF1b constitutive expression for qPCR
MiEF1b-r1	TTTGGCAATAACTCCAGGCTTTTTAG	MiEF1b constitutive expression for qPCR
MiIDA-f2	TGCTTTTATCTGTCTCAATTGTGGA	MiIDL1 qPCR
MiIDA-r2	AATCTATCGGCCGGGACCTG	MiIDL1 qPCR
MiIDL2-f1	TCTGTCTCAATTGTGGATGCAG	MiIDL2 qPCR
MiIDL2-r1	ATTCAATCAGCCTGGATGACCTGGAGG	MiIDL2 qPCR
GUS_RNAi-f1	CTCCTACCGTACCTCGCATTAC	GUS qPCR
GUS_RNAi-r1	CGCTTCGAAACCAATGCCTAAAG	GUS qPCR
KAN1	CTGTCATCTCACCTTGCTCC	NPTII qPCR
KAN2	GCCAAGCTCTTCAGCAATATC	NPTII qPCR
MiIDL1-F1N	CCGctcgagAAAATGTTTTATTCAATTAAAAATTTAATTAATT	Full-length MiIDL1 overexpression construct
MiIDL1-R1N	GCtctagaCTATCGGCCGGGACCTGGAA	Full-length MiIDL1 overexpression construct
MispIDL1-F1N	CCGctcgagAAAATGATTAAAGGAGTTCCACCTAATAGTG	No Signal Pep MiIDL1 overexpression construct
MispIDL1-R1N	GCtctagaCTATCGGCCGGGACCTGGAACTTTA	No Signal Pep MiIDL1 overexpression construct
GUS_RNAi_XBA_f1	ACAAACtctagaACTTTACTGGCTTTGGTCGTC	GUS RNAi construct
GUS_RNAi_HIND_r1	CCACATaagcttGCTTGGGTGGTTTTTGTCAC	GUS RNAi construct
GUS_RNAi_XHO_f1	ACAAACctcgagACTTTACTGGCTTTGGTCGTC	GUS RNAi construct
GUS_RNAi_ECOR_r1	CCACATgaattcGCTTGGGTGGTTTTTGTCAC	GUS RNAi construct
MiIDL1_RNAi_XBA_f1	AATTTtctagaAGACATACCGCCAAAAATGTTTTATTC	MiIDL1 RNAi construct
MiIDL1_RNAi_HIND_r1	TTTTTTaagcttCCTTTATATATTTATTCCAAAAATTACTAA	MiIDL1 RNAi construct
MiIDL1_RNAi_XHO_f1	AATTTctcgagAGACATACCGCCAAAAATGTTTTATTC	MiIDL1 RNAi construct
MiIDL1_RNAi_ECOR_r1	TTTTTTgaattcCCTTTATATATTTATTCCAAAAATTACTAA	MiIDL1 RNAi construct
